# Enhancing semantical text understanding with fine-tuned large language models: A case study on Quora Question Pair duplicate identification

**DOI:** 10.1371/journal.pone.0317042

**Published:** 2025-01-10

**Authors:** Sifei Han, Lingyun Shi, Fuchiang (Rich) Tsui

**Affiliations:** 1 Department of Biomedical and Health Informatics, Tsui Laboratory, Children’s Hospital of Philadelphia, Philadelphia, PA, United States of America; 2 Department of Anesthesiology and Critical Care, Children’s Hospital of Philadelphia, Philadelphia, PA, United States of America; 3 Department of Anesthesiology and Critical Care, University of Pennsylvania Perelman School of Medicine, Philadelphia, PA, United States of America; 4 Department of Biostatistics, Epidemiology and Informatics, University of Pennsylvania Perelman School of Medicine, Philadelphia, PA, United States of America; Beijing University of Technology, CHINA

## Abstract

Semantical text understanding holds significant importance in natural language processing (NLP). Numerous datasets, such as Quora Question Pairs (QQP), have been devised for this purpose. In our previous study, we developed a Siamese Convolutional Neural Network (S-CNN) that achieved an F1 score of 82.02% (95% C.I.: 81.83%-82.20%). Given the growing attention toward large language models (LLMs) like ChatGPT, we aimed to explore their effectiveness in text similarity tasks. In this research, we leveraged 5 pretrained LLMs, conducted various fine-tuning approaches (prompt engineering, n-shot learning, and supervised learning using the low-rank adaptation [LoRA]), and compared their performance using F1 score. To ensure a fair comparison, we followed our previous study’s design and dataset by employing a 10-fold cross-validation for supervised model training and evaluation. Additionally, we conducted a secondary study by introducing a recent larger LLM with 70B parameters and comparing it with the 7B model using the GLUE benchmark, and both models were finetuned with the corpus. The fine-tuned LLaMA model with 7B parameters (qLLaMA_LoRA-7B) using 100,000 QQP corpus yielded the best results, achieving an F1 score of 84.9% (95% C.I.: 84.13%-85.67%), which outperformed the Alpaca_LoRA-65B (finetuned based on LLaMA-65B) (F1: 64.98% [64.72%-65.25%]; P<0.01) and had a 3% improvement compared to our previously published best model, S-CNN. The finetuned LLaMA3.1-70B (qLLaMA3.1_LoRA-70B) with 70B parameters (F1: 74.4%) outperformed the qLLaMA_LoRA-7B (F1: 71.9%) using the GLUE benchmark. The study demonstrated an effective LLM finetuning framework, which highlights the importance of finetuning LLMs for improved performance. Our task-specific supervised finetuning demonstrated improved LLM performance compared to larger pretrained models with or without n-shot learning; moreover, finetuning a larger LLM further improved performance compared to finetuning a smaller LLM. Our LLM-based finetuning framework may potentially improve various document similarity tasks, such as matching resumes with job descriptions, recommending subject-matter experts, or identifying potential reviewers for grant proposals or manuscript submissions.

## 1. Introduction

Semantical text understanding is a fundamental aspect of natural language processing (NLP) tasks, enabling machines to comprehend and extract meaning from textual data. It plays a pivotal role in various applications, including question pair classification [1], document similarity measurement [2], and content matching [3]. In this study, we focus on the duplicate identification of Quora Question Pair (QQP) [4] and propose a novel approach leveraging the power of large language models (LLMs) [5] for improved performance.

Quora, a well-known question-and-answer platform, grapples with the issue of pinpointing duplicate questions to improve user experience and avoid repetition. Conventional methods used for duplicate (similarity) detection commonly rely on heuristics and manually curated features [6,7], which restricts their ability to truly understand the subtle semantic aspects of natural language. As a result, it becomes imperative to investigate more sophisticated approaches that can harness the natural language comprehension abilities offered by large language models.

In our previous work, we developed the Siamese convolutional neural network (S-CNN) in conjunction with Bidirectional Encoder Representations from Transformer (BERT) model [8], which achieved promising results with an F1-score of 82.02% on the Quora Question Pair dataset.

With the advent of large language models (LLMs) like ChatGPT [9] by OpenAI and Gemini by Google, they have shown remarkable capabilities in natural language understanding. However, researchers face challenges using commercial LLMs, such as their black-box models that may not be updated by researchers and data being commonly processed outside researchers’ facilities, which may raise data security and confidentiality concerns. Recent open-sourced LLMs (using 10^9^–10^11^ parameters), e.g., Large Language model Meta AI (LLaMA) [10] developed by Meta AI and Mistral models by Mistral AI [11], shed light to address the challenges researchers are facing. However, it remains unknown to what extent such open-sourced LLMs can further improve previously developed language models (using approximately 10^8^ parameters) such as our previously developed S-CNN in conjunction with the BERT model in question pair classification tasks.

### 1.1 Research objectives

The primary objective of this research is to assess the effectiveness of open-sourced LLaMA models in improving text similarity tasks. Specifically, we aimed to investigate the potential of LLaMA when fine-tuned using the low-rank adaptation (LoRA) technique. This study seeks to advance our understanding of how LLMs can enhance text similarity measurements, with a particular focus on the QQP dataset. Additionally, we aimed to achieve a better classification performance, evaluated with the F1-score, and determined the extent of improvement compared to our prior best-performing model [8]. Furthermore, we explored prompt engineering and few-shot prompting approaches on LLMs and compared performance between different approaches.

The contributions of this work include demonstrating the potential of large language models, such as LLaMA-7B, in improving question pair classification tasks, and fine-tuning is essential for specific tasks. Additionally, our approach shows promise in enhancing document similarity measurement, potentially enabling practical applications in fields such as resume matching [12], expert finding [13], request-for-proposal recommendations [14], and assisting editors in identifying potential reviewers.

The main contributions of this paper are summarized as follows:

1. Exploring five pretrained open-sourced LLMs: We undertook a comprehensive exploration of large language models, experimenting with various approaches to identify effective methods for downstream tasks. To the best of our knowledge, this is the first study that applied LLMs to similarity comparison in the QQP dataset.
2. Optimizing LLMs using task-specific finetuning framework: We developed a framework to fine-tune LLMs and evaluated the performance using the existing QQP corpus and an open benchmark dataset. We also compared a finetuned LLM to our previous best-performing model, the Siamese-CNN model [8].
3. Evaluating Prompt Engineering and n-shot prompting approaches: Our research underscores the role of prompt engineering when utilizing large language models, and evaluated performance between different n-shot prompting approaches.
4. Comparing finetuned LLMs using the General Language Understanding Evaluation (GLUE) Benchmark [15]: We compared two fine-tuned LLMs (qLLaMA-LoRA-7B and qLLaMA3.1_LoRA-70B) using the GLUE Benchmark, a common standard to assess model performance using QQP test data.

### 1.2 Related work

This section briefly describes our previous best model and introduces the challenges associated with this task.

#### 1.2.1 Siamese Convolutional Neural Network (S-CNN)

In our previous work, we developed an S-CNN model [8] to identify duplicate Quora Question Pairs. This approach utilized a Siamese architecture [1,16,17] consisting of two parallel convolutional neural networks (CNNs) [18–21] with shared weights. Each network independently processed one question from the pair, enabling the model to learn representations that captured the semantic similarities [22,23] and differences between the questions. To enhance processing, attention mechanisms [24–26] were incorporated to focus on different sections of the question text based on their significance when determining similarity. The outputs of the Siamese networks were then passed through a similarity measure, generating a similarity probability and then determining whether the question pair was a duplicate or not.

#### 1.2.2 LLaMA models

MetaAI has developed several large language models (LLaMA) that use the Transformer architecture to process natural language on a vast scale [10]. LLaMA models have been trained on an extensive corpus of text and code, which enhances its capabilities in generating, understanding, and answering queries in natural language. The foundation LLaMA models (first introduced in 2023) range from 7B to 405B (LLaMA3.1) parameters, and the models were trained on trillions of tokens. The LLaMA-13B outperformed GPT-3 (175B) [27] on most benchmarks, and LLaMA-65B is competitive with models like Chinchilla-70B [28] and PaLM-540B [29]. MetaAI has released the models to the research community.

#### 1.2.3 GLUE benchmark

The General Language Understanding Evaluation (GLUE) benchmark [15], developed by New York University and DeepMind researchers, is pivotal in assessing machine learning models across a spectrum of language understanding tasks, such as sentiment analysis, question answering, and textual entailment. It includes datasets like the Quora Question Pairs (QQP), allowing models like ours to be rigorously evaluated on their ability to generalize across various linguistic tasks. GLUE’s structure promotes advancements in NLP by testing models, particularly those based on transformative architectures like BERT [30] and GPT [27], creating a competitive yet collaborative research environment.

Recent advancements in NLP are primarily fueled by transformer-based architectures such as BERT, GPT, and Google’s T5 [31]. BERT enhances understanding through bidirectional training, while GPT-3, with its vast parameter scale, excels in generating contextually relevant text without specific tuning. T5 simplifies processing by treating all language tasks as text-to-text conversions, showcasing the shift towards versatile, adaptable models that streamline deployment across different applications, thus driving significant progress in the field.

#### 1.2.4 Challenges and limitations of current NLP models

While our previously published Siamese-CNN model achieved promising results with an F1-score of 82.02% on the Quora Question Pair dataset [32], there still exist challenges and limitations.

- **Natural Language Understanding**

The Siamese-CNN approach, which utilizes feature representations for semantical understanding, faces challenges and limitations in capturing the subtleties and complexities of language, potentially compromising the accuracy of tasks like question pair classification; such limited context understanding limits its generalizability in different applications and results in many model variations for specific tasks, e.g., bioBERT [33], clinicalBERT [34], and strokeBERT [35]. In contrast, advancements in natural language understanding have been exemplified by the emergence of large language models, such as OpenAI’s ChatGPT-3 [9] with 175 billion parameters. These models significantly outperform smaller-scale models like the BERT model with 26 million parameters, particularly in grasping contextual nuances and semantic intricacies, thus promising substantial improvements across a range of NLP applications [36,37].

- **Performance Improvement**

Given the limitations of our previous approach and the growing interest in LLM, we aimed to explore the potential of fine-tuning a large language model for question pair duplicate identification. This exploration presented an opportunity to achieve further improvements in performance and enhance the accuracy of classification.

In this study, we hypothesized that by fine-tuning a large language model, we could harness its language understanding abilities and improve the performance of duplicate question-pair identification. The key contributions of this study include 1) addressing these challenges and limitations of current NLP models by developing a framework to optimize large language models and 2) comparing their performance using different optimization steps in our QQP corpus and the standard GLUE benchmark. Our work leverages the strengths of large language models, focusing on their natural language understanding abilities to improve question pair classification tasks.

## 2 Materials and methods

We first describe the QQP corpus used in this study followed by the introduction of the low-rank adaptation (LoRA) algorithm [Section 2.3] [38], which enables finetuning an LLM by updating a small fraction of the model’s parameters to shorten the training time and save resources.

### 2.1 Quora Question Pair (QQP) corpus

In this study, we used the Quora Question Pairs (QQP) dataset as our study corpus, shared via a Kaggle competition, consisting of a publicly available annotated dataset with 404,290 question pairs. The dataset contains 255,027 pairs (63.08%) labeled as ’0’ (indicating non-duplicate or dissimilar pairs) and 149,263 pairs (36.92%) marked as ’1’ (indicating duplicate or similar pairs). These labels were meticulously assigned by human experts.

### 2.2 Classification task/outcome

The task for the language models in this study is to provide a binary classification on whether the question pair of interest is a duplicate (marked as “yes”) or non-duplicate (marked as “no”) pair.

### 2.3 The LoRA algorithm for updating parameters of a large language model

Traditionally, fine-tuning a language model involves updating all parameters of the pre-trained model. However, this can be time-consuming and costly for researchers and companies. To address this, we employed the Low-Rank Adaptation (LoRA) [38] for effectively updating parameters in an LLM. LoRA maintains the weights of the pre-trained model while introducing trainable low-rank decomposition matrices. By freezing the pre-trained model weights and incorporating trainable low-rank matrices into each layer, LoRA significantly reduces the total number of trainable parameters. This reduction in parameters makes it feasible to train LLMs with significantly fewer computational resources. The following equation summarizes the LoRA algorithm where *h* represents the output of a given network layer, *x* is the input from the previous layer, $W_{0}\in R^{d\times k}$ represents the majority of weights in the current layer, *k* is the input dimension from the previous layer, *d* represents the output dimension; A and B are two low-rank decomposed trainable matrices, $A\in R^{d\times r}$ and $B\in R^{r\times k}$, where *r* denotes the pre-determined rank. During the forward pass with an input *x*, the equation is as follows:

$$h=W_{0}x+\Delta Wx=W_{0}x+ABx,A\in R^{d\times r},B\in R^{r\times k}$$

During training, *W*_0_ remains frozen (unchanged) and does not receive gradient updates, while *A* and *B* are updated. By selecting a rank, the memory consumption can be effectively reduced as there is no need to store the optimizer states for the large frozen matrix.

### 2.4 LLM Optimization Framework

In this framework, we describe three steps to optimize a large language model: prompt engineering, n-shot promoting, and supervised fine-tuning.

#### 2.4.1 Prompt engineering

Prompt engineering involves crafting input prompts that effectively guide LLMs to perform specific tasks. This technique includes designing clear instructions, embedding relevant context, or using structured templates to maximize the model’s output quality without retraining. The goal is to optimize the LLM’s performance by adjusting how information is presented, enabling it to handle various tasks effectively with minimal data or fine-tuning. Effective prompt engineering requires iterative experimentation to determine the best structure for achieving desired results. In this step, providing examples (n-shot prompting) is an option (see the next section).

#### 2.4.2 N-shot prompting

**Fig 1:**
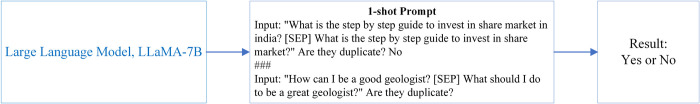
One-shot prompt for the Large Language model Meta AI (LLaMA) model with 7 billion parameters.

N-shot prompting refers to providing an LLM with ‘n’ labeled examples in the prompt to help it perform a specific task. The number ‘n’ indicates how many examples are shown to the model before it generates a response for a new, unseen input. This technique leverages the model’s pre-trained knowledge, using minimal examples to enhance performance on tasks without needing extensive retraining. Fig 1 shows the process of 1-shot prompting (learning), a crucial aspect of our few-shot prompting [39–41] experiments, which aim to optimize the model’s ability to perform tasks using only limited input. The flowchart highlights the steps involved in leveraging such examples to guide the model.

In our specific use case, each 1-shot prompt [42] consists of a concatenation of two questions, separated by the special token [SEP], followed by the query “Are they duplicates?” The LLaMA model is then tasked with generating a response either “Yes” or “No” based on the information provided. -represents this process emphasizes the role of prompt engineering in effectively guiding the model’s reasoning with minimal data. To enable scalability in few-shot prompting, we employ the use of “###” as a separator between different examples within the prompt, allowing the model to handle multiple samples efficiently. This approach ensures that the model remains flexible, processing various datasets with different numbers of examples while maintaining accuracy and consistency. With this approach, the model can acquire new knowledge quickly and perform better on the downstream task with limited labeled data.

#### 2.4.3 Supervised fine-tuning (SFT)

Supervised finetuning is the process of further training a pre-trained LLM on a specific dataset where both input data and corresponding output labels are provided. This allows the model to adapt its knowledge to perform well on a particular task or domain. Unlike n-shot prompting, which relies on few examples embedding in prompts and does not change the parameters of the LLM, supervised fine-tuning involves training the model on larger labeled datasets and subsequently updates the parameters of the LLM, allowing the LLM to make more accurate and reliable predictions.

**Fig 2:**
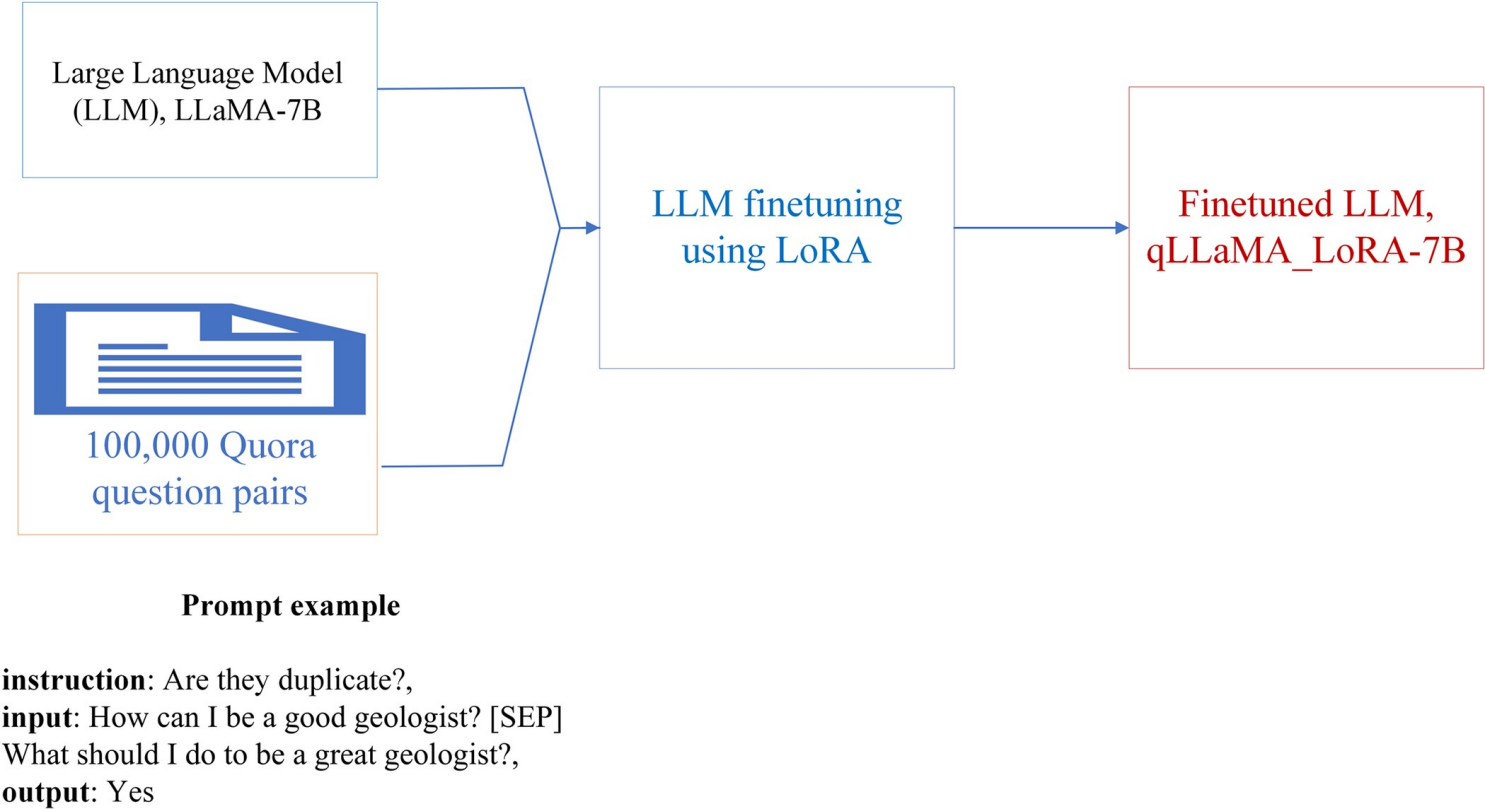
The fine-tuning flowchart for the qLLaMA_LoRA-7B model by updating the pre-trained LLaMA-7B model’s parameters using the Low-Rank Adaptation (LoRA), a supervised learning algorithm, from 100,000 Quora question pairs. LLaMA-7B: Large Language model Meta AI with 7 billion parameters.

In this step, we took a pre-trained model, such as LLaMA-7B, and fed the labeled examples from the QQP dataset. These labeled examples consist of inputs (e.g., question pairs) and the corresponding outputs (e.g., “Yes” or “No”). Through iterative training, the model adjusts its internal parameters to minimize the difference between its predicted outputs and the ground truth provided in the dataset. With this approach, it makes model highly task-specific, improving performance on specialized tasks like duplicate identification [8], sentiment analysis [43]. Finetuning helps to address limitations in the general pre-trained model by training it on domain-specific terminology and nuances. This process generally results in better performance than N-shot prompting (learning), as the model becomes more finely tuned to the specific dataset it will encounter in production. We named the model qLLaMA-7B-LoRA as the finetuned LLaMA-7B applied to QQP through the supervised finetuning using LoRA (Fig 2).

### 2.5 Eight classification models: three state-of-the-art NLP Models and five LLMs

In the main study, we compared and evaluated 8 models: three previously published state-of-the-art NLP models (Simple Contrastive Sentence Embeddings (SimCSE) [44], a contrastive learning-based approach MirrorBERT [45], and S-CNN), four pre-trained LLMs (Alpaca_LoRA-7B, Alpaca_LoRA-65B, LLaMA-7B, LLaMA-33B) and one supervised fine-tuning LLM (qLLaMA-7B-LoRA). We further compared the performance of 1-shot and 5-shot prompts between LLaMA-7B and LLaMA-33B: LLaMA-7B_1shot, LLaMA-7B_5shot, LLaMA-33B_1shot, and LLaMA-33B_5shot. SimCSE and MirrorBERT are contrastive learning-based models, while S-CNN utilizes an auto-encoder with a Siamese-CNN network; the three models, having the number of variables ranging between 10^7^ and 10^8^, are considered as baseline models for the textual semantic similarity task. The four pre-trained LLMs having the number of variables ranging between 10^9^ to 10^10^, are described below:

- LLaMA-7B is a highly efficient model from Meta AI, designed to perform well with fewer resources while maintaining strong performance across various NLP tasks. It has 7 billion parameters and trained on 1.4 trillion tokens from a curated dataset, which consists of about 1 million unique documents [10]. It strikes a balance between computational efficiency and robust task execution.
- LLaMA-33B is a larger variant of the LLaMA family, consisting of 33 billion parameters and trained on 1.4 trillion tokens same as LLaMA-7B [10]. This model offers enhanced capacity and performance.
- Alpaca_lora_7B is a fine-tuned version of LLaMA using LoRA which enables more efficient training with limited computational resources, it retains LLaMA’s base architecture but is designed to excel in instruction-based tasks through additional fine-tuning on a dataset of 52,000 instruction-following examples generated by OpenAI’s text-davinci-003 [46].
- Alpaca_lora_65B is a larger version of Alpaca, with 65 billion parameters, offering improved contextual understanding and handling of complex language tasks. It was fine-tuned using the same instruction-following dataset as Alpaca_lora_7B model.

### 2.6 Model training and evaluation

We implemented a 10-fold cross-validation [47] strategy for training and assessing the fine-tuned LLaMA model. The dataset was initially divided into ten stratified folds. Subsequently, a model was trained using nine of the 10 folds and then evaluated using the remaining fold as a hold-out dataset. The process was repeated 10 times. In our previous study [8], we established a benchmark by conducting a comprehensive comparison of diverse deep-learning approaches, ultimately identifying the S-CNN as the superior model. To ensure fairness in our comparisons, we applied the identical fold splits that were previously created in our previous study [8] and conducted experiments on the same machine when assessing the performance of large language models. Due to the complexity of LLMs, we performed stratified sampling of each training fold from 363,861 to 100,000 question pairs (comprising 36,920 similar and 63,080 dissimilar pairs). We kept the same size of each test fold for performance evaluation.

#### 2.6.1 LLM fine-tuning hyperparameters

For the fine-tuning process, we used a batch size of 128 and conducted ten epochs and the additional hyperparameters adopted from the Alpaca model, which were maintained at their default settings, include a learning rate of 3e-4, lora_r set to 8, lora_alpha set to 16, and a lora_dropout rate of 0.05. In the inference stage, the default settings were retained with a “temperature” (a hyperparameter used in softmax calculation during the generation process. A higher temperature value leads to more randomness in the selection of words, potentially creating more diverse but less predictable text.) of 0.1, top_p (nucleus sampling, a method used in text generation where the model only considers a subset of the vocabulary with the highest probabilities whose cumulative sum is less than or equal to the specified p value) of 0.75, top_k (top_k sampling, model only considers the top k most probable next words at each step of the generation) of 40, and num_beams (beam search, number of sequences considered at each step of the generation process) of 4.

### 2.7 Model evaluation metrics

Evaluation metrics for NLP models in this study include precision, recall/sensitivity, accuracy, and F1-score [48], defined below: $Precision=\frac{TP}{TP+FP},\;Recall=\frac{TP}{TP+FN},\;F1\;score=2*\frac{Precision*Recall}{Precision+Recall}$

where *TP*, *FP*, and *FN* are true positive, false positive, and false negative, respectively. The ideal classifier has *precision* and *recall* equal to one, which means *FP* and *FN* are zero. F1-score is the harmonic mean of *precision* and *recall*.

### 2.8 Secondary study: Fine-tuning and evaluating the LLaMA3.1-70B model

With the release of more advanced, larger, and robust foundation models, we extended our main analysis of 10 models by fine-tuning another recent pre-trained model, LLaMA3.1-70B, with 70 billion parameters; the fine-tuned model, qLLaMA3.1-LoRA-70B, was then evaluated in an external dataset, on the GLUE Benchmark. Using the same approach and prompts described in the main study, we fine-tuned the LLaMA3.1-70B model for the QQP task through supervised training using LoRA. Due to the complexity of LLMs, we performed a stratified sampling of 100,000 question pairs from one of the training folds comprising 363,861 question pairs as described in Section 2.6. The supervised fine-tuning was based on the 100,000 pairs to adjust the model’s weights and optimize its performance on the QQP task. After completing the fine-tuning process, the fine-tuned qLLaMA3.1-LoRA-70B model was then externally validated using the GLUE Benchmark dataset. The results generated from the LLaMA3.1-70B model were then submitted to the official GLUE Benchmark platform for performance scoring. This submission allowed us to obtain comparative performance metrics, providing a clearer understanding of how the fine-tuned LLaMA3.1-70B model fares against other models in terms of handling the QQP task.

## 3. Results

### 3.1 Main study: Comparison between three state-of-the-art NLP models and five LLMs

**Table 1** summarizes classification performance of eight models, three state-of-the-art NLP models (SimCSE, MirrorBERT, and S-CNN) and five LLMs (qLLaMA_LoRA-7B, Alpaca_LoRA-7B, Alpaca_LoRA-65B, LLaMA-7B, and LLaMA-33B), with 1-shot and 5-shot promptings between LLaMA-7B and LLaMA-33B. The fine-tuned LLaMA-7B model using LoRA (qLLaMA_LoRA-7B) demonstrated the best F1 score (84.9% [95% CI: 84.13–85.67]), precision (83.64% [95% CI: 81.1–86.18]), and accuracy (88.67% [95% CI: 88.35–88.99]) with statistical significance (P < .05), compared to other nine NLP models using t-test. SimCSE had the best recall of 99.78 (95% CI, 99.73–99.83). The fine-tuning procedure was performed on a server with a single Nvidia Tesla V100 graphics processing unit (GPU) with 32GB, which took approximately 66 hours for one-fold training. The prompting of LLaMA-7B, Alpaca_LoRA-7B, and Alpaca_LoRA-65B models were tested on another server with a single Nvidia Tesla A100-80GB GPU. The LLaMA-33B model was tested on the third server with 4 A100-80GB GPUs given the MetaAI script requirement.

**Table 1:** Model comparison for question pairs detection using 10-fold cross validation.

Model	F1 [%] (95% C.I.)	Precision [%] (95% C.I.)	Recall [%] (95% C.I.)	Accuracy [%] (95% C.I.)
**SimCSE**	61.22 (61.15–61.29)	44.16 (44.09–44.23)	**99.78** **(99.73–99.83)**	53.34 (53.21–53.46)
**MirrorBERT**	60.14 (60.05–60.22)	43.07 (42.98–43.16)	99.60 (99.54–99.66)	51.25 (51.07–51.42)
**S-CNN**	82.02 (81.83–82.20)	82.18 (81.99–82.38)	81.88 (81.60–82.17)	83.32 (83.17–83.47)
**qLLaMA_LoRA-7B**	**84.90** **(84.13–85.67)**	**83.64** **(81.10–86.18)**	86.43 (82.39–90.46)	**88.67** **(88.35–88.99)**
**Alpaca_LoRA-7B**	4.67 (4.39–4.95)	34.13 (28.41–39.84)	2.50 (2.38–2.63)	62.40 (61.86–62.95)
**Alpaca_LoRA-65B**	64.98 (64.72–65.25)	58.59 (58.35–58.83)	72.94 (72.56–73.33)	70.96 (70.75–71.16)
**LLaMA-7B**				
** 0-shot prompting**	0.22 (0.21–0.23)	33.45 (30.12–36.78)	0.11 (0.10–0.11)	0.18 (0.17–0.18)
**1-shot prompting (LLaMA-7B_1shot)**	20.90 (20.54–21.27)	38.13 (37.40–38.86)	14.40 (14.14–14.65)	59.77 (59.54–60)
**5-shot prompting (LLaMA-7B_5shot)**	28.03 (27.61–28.45)	40.78 (40.19–41.38)	21.36 (21–21.72)	59.52 (59.27–59.76)
**LLaMA-33B**				
** 0-shot prompting**	0.41 (0.39–0.42)	35.66 (33.67–37.65)	0.20 (0.19–0.21)	0.34 (0.32–0.35)
**1-shot prompting (LLaMA-33B_1shot)**	31.77 (31.31–32.22)	37.27 (36.84–37.70)	27.68 (27.19–28.16)	56.10 (55.87–56.33)
**5-shot prompting (LLaMA-33B_5shot)**	49.39 (48.92–49.85)	50.40 (50–50.81)	48.41 (47.84–48.98)	63.36 (63.08–63.65)

The boldfaced numbers represent the best performance across models. SimCSE: Simple Contrastive Sentence Embeddings, a contrastive learning-based model for sentence embedding; MirrorBERT: a contrastive learning-based model designed to improve sentence representations; S-CNN: Siamese Convolutional Neural Network for text similarity tasks; qLLaMA_LoRA-7B: Quantized Low-Rank Adaption of LLaMA with 7 billion parameters; Alpaca_LoRA-7B/65B: Finetuned model by using LoRA based on LLaMA-7B/65B model. LLaMA-7B/33B_1shot/5shot: LLaMA model with 7/33 billion parameters using 1-shot or 5-shot prompting.

Few-shot prompting was particularly effective for larger models, showing notable improvements between the 1-shot and 5-shot settings. For example, the LLaMA-7B model’s F1 score was increased by 34.1% from 20.90% (95% CI: 20.54%-21.27%) in the 1-shot prompting to 28.03% (95% CI: 27.61%-28.45%) in the 5-shot prompting. Additionally, the larger model LLaMA-33B had further improvement using few-shot learning compared to the smaller model LLaMA-7B, i.e., LLaMA-33B’s F1 score was increased by 55.5% from 1-shot 31.77% (95% CI: 31.31%-32.22%) to 5-shot 49.39% (95% CI: 48.92%-49.85%).

### 3.2 Secondary study: External validation using the GLUE benchmark dataset

**Table 2** summarizes the external validation performance results of two supervised fine-tuned LLaMA models with 7B and 70B parameters: qLLaMA_LoRA-7B and qLLaMA3.1_LoRA-70B. The qLLaMA3.1_LoRA-70B outperformed the smaller model qLLaMA_LoRA-7B. The qLLaMA3.1-LoRA-70B model was trained on a server with four Nvidia A100-80GB GPUs, which took approximately 18 hours for model fine-tuning.

**Table 2:** External validation results between two fine-tuned models (qLLaMA_LoRA-7B and qLLaMA3.1_LoRA-70B) based on GLUE benchmark.

Model	F1 (%)	Accuracy (%)
qLLaMA_LoRA-7B	71.9	89.1
qLLaMA3.1_LoRA-70B	**74.4**	**90.3**

qLLaMA_LoRA-7B: the fine-tuned LLaMA-7B model using supervised learning based on LoRA. qLLaMA3.1_LoRA-70B: the fine-tuned LLaMA3.1-70B model using supervised learning based on LoRA. No confidence interval was obtained since the analyzed performance results were computed by the GLUE benchmark.

## 4. Discussion

In this study, we tested our hypothesis on whether an optimized open-sourced LLM, e.g., qLLaMA_LoRA-7B, through supervised learning using Quora-question pairs, can improve similarity classification performance compared to the current state-of-the-art language models (simCSE, mirrorBERT, S-CNN) and out-of-the-box pre-trained LLMs. At the outset of this study, LLaMA models stood out as the leading open-source model in terms of both performance and accessibility for research purposes. The supervised fine-tuned model, qLLaMA_LoRA-7B, performed best compared to the other seven models, including unsupervised learning, zero-shot, one-shot, and 5-shot prompting on varying sizes of LLMs; it had an improved F1-score of 84.90% on the QQP dataset compared to the three state-of-the-art language models with statistical significance (P < .01). This improvement highlights the potential of LLaMA in enhancing the task of classifying question pairs and showcases the benefits of leveraging LLMs for semantical text understanding. Notably, the fine-tuned qLLaMA_LoRA-7B model outperformed larger LLMs, including those with 33B and 65B parameters, which suggests a smaller fine-tuned LLM can potentially perform better than those larger LLMs without supervised fine-tuning given that supervised fine-tuning allowed the model to adapt to the specific task of question-pair duplicate detection.

The S-CNN baseline model employed a Siamese network structure combined with convolutional neural networks, necessitating more intricate architectural design, and achieved an F1-score of 82.02%. In contrast, the out-of-the-box Alpaca_LoRA-65B model, while demonstrating its inherent power, had a lower F1-score of 64.98%, suggesting that a pretrained LLM might not be fully optimized for the specific task compared to current state-of-the-art NLP models. On the other hand, the qLLaMA_LoRA-7B, a fine-tuned version of the LLaMA-7B model tailored for the Quora Question Pairs dataset, significantly improved performance with an F1-score of 84.9%.

The disparity in performance between out-of-the-box Alpaca_LoRA-7B and Alpaca_LoRA-65B is stark, with F1 scores of 4.67% and 64.98%, respectively. This significant difference highlights the impact of pretrained model size on task performance. The 7B model, with its relatively limited parameter count, struggled to capture the complex patterns necessary for accurate text similarity classifying. In contrast, the 65B model, with its vastly larger parameter space, demonstrated an improved ability to comprehend and process linguistic nuances, leading to a much higher F1 score. This suggests that for certain NLP tasks, particularly those requiring nuanced understanding, larger models may offer substantial advantages.

Few-shot prompting can potentially improve classification performance in the same model. Notably, the pre-trained LLaMA-7B with 0-shot prompting had only 0.24% F1-score, whereas LLaMA-7B_1shot and LLaMA-7B_5shot had 20.9% and 28.03% F1scores (87 and 117 times higher than the 0-shot performance), respectively. Similarly, the pre-trained LLaMA-33B had improved F1 scores of 0.41%, 31.77%, and 49.39% using 0, 1 and 5 shots of prompting, respectively. The 1-shot and 5-shot F1 scores were x and y times higher than the 0-shot F1 score. These findings underscore the effectiveness of few-shot prompting, especially when rapid outcome improvement is needed without running LoRA. We attribute the improved performance through n-shot prompting is due to learning of question-answer patterns and additional contextual guidance [27].

A larger LLM can potentially benefit more from few-shot prompting compared to a smaller LLM. Between the two pretrained models (LLaMA-7B and LLaMA-33B), after 5-shot prompting, the larger model LLaMA-33B had best F1 score (49.39% vs. 28.03%) with statistical significance, P < .05. Also, compared to LLaMA-7B, the LLaMA-33B model had a much higher F1 score improvement rate between 1-shot and 5-shot, i.e., 55.5% vs. 34%. It can be attributed by the flexibility and the number of parameters of a larger LLM.

Larger LLMs generally performed better than smaller LLMs at 0-shot prompting. Larger models like Alpaca_LoRA-65 and LLaMA-33B, significantly outperformed their counterpart smaller models, Alpaca_LoRA-7B and LlaMA-7B. It suggests that the same task could be achieved with improved performance given the large model’s greater capacity for learning and generalizing. The larger number of parameters enables the bigger models to capture more intricate and nuanced patterns in the data, enabling them to reach a higher level of abstraction and understanding.

In Section 1.2.3, we noted that the QQP dataset is part of the GLUE benchmark. To better understand the performance of the qLLaMA_LoRA-7B model compared to other approaches, we evaluated it on this dataset. Our model achieved an F1-score of 71.9% and an accuracy of 89.1%, which were slightly lower (4.5% and 1.8%) than the scores of the leading team (Microsoft) [49], respectively. However, it is worth noting that human baselines were 59.5% F1-score and 80.4% accuracy.

A larger supervised finetuned (SFT) LLM performed better than a smaller SFT LLM. As outlined in the secondary study, we examined the improvements across different model generations. Our SFT qLLaMA3.1_LoRA-70B outperformed the qLLaMA-LoRA-7B by achieving an F1-score of 74.4% (vs. 71.9%) and an accuracy of 90.3% (vs. 89.1%). The 70B model closely matched the top-ranked 8th model on the leaderboard (F1-score of 74.7%, accuracy of 90.6%) with very close performance. As both LLMs were trained based on 100K pairs vs. others that were trained with the full set of 255K pairs, we believe our SFT LLMs could have potentially increased performance through additional training pairs.

### 4.1 Limitations

Our study has the following limitations. First, we only performed few-shot prompting on two LLMs instead of all 5 LLMs. It was due to available hardware resources. Since the two Alpaca models were derived from LLaMA models, we believe the impact without considering the Alpaca n-shot prompting is minimal. Second, we did not perform prompt engineering to compare multiple prompt questions, e.g., “Are the two questions similar?”, for few-shot prompting experiments. Literature shows that while few-shot prompting holds significant potential for enhancing model performance, the effectiveness of the approach heavily depends on well-crafted prompts. Further investigations with refined and optimized prompts could be considered as it may yield more accurate and robust outcomes, providing a clearer picture of the model’s true capabilities in few-shot prompting scenarios. Third, to reduce the computational load, we did not use the full 255K pairs for SFT LLMs but rather downsampled the full annotated dataset to 100,000 pairs. We may consider to train the LLMs with the full QQP dataset. Finally, we did not perform 10-fold cross-validation on the qLLaMA3.1_LoRA-70B to provide a more comprehensive evaluation with the 8 models due to limited resources.

### 4.2 Future work

The research presented in this study introduces opportunities for future investigation and advancement. The following paths can be pursued to expand our work and progress further.

1. Scaling and Efficiency: As LLMs continue to grow in size and complexity, research efforts can focus on improving their scalability and computational efficiency. Techniques such as model distillation, pruning, and model compression/quantization [50–53] can be explored to make these models more accessible and practical for real-world applications.
2. Prompt Engineering: LLMs are highly sensitive to context, and identical semantic inputs can yield different outcomes. To optimize the performance of these models, experimenting with various prompt formats, styles, and the inclusion of different types of information will be beneficial.

## 5. Conclusions

In this study, we compared the performance of eight NLP models for document similarity classification, including three current-state-of-the-art NLP models (SimCSE, MirrorBERT, and S-CNN) and five open-sourced large language models. A large language model with a smaller size (e.g., 7B) with supervised fine-tuning for a specific task can potentially perform better than those models with a much larger size (e.g., 65B) and current state-of-the-art text-similarity classification models. A finetuned larger LLM is likely to outperform a smaller finetuned LLM. Our work not only demonstrates its potential in measuring document similarity, but also potentially enables broad applicability in various natural language understanding tasks. By accurately capturing semantic nuances and language patterns, an NLP model can contribute to tasks such as aligning resumes with job descriptions, locating experts, recommending RFPs to researchers of interest, and aiding editors in finding suitable reviewers.
